# Immunomodulation, Toxicity, and Therapeutic Potential of Nanoparticles

**DOI:** 10.3390/biotech11030042

**Published:** 2022-09-09

**Authors:** Ashutosh Pandey, Abhinava K. Mishra

**Affiliations:** 1 Department of Molecular and Human Genetics, Baylor College of Medicine, Houston, TX 77030, USA; 2 Molecular, Cellular and Developmental Biology Department, University of California Santa Barbara, Santa Barbara, CA 93106, USA

**Keywords:** toxicity, immunotoxicity, immunomodulation, nanoparticles, immunotherapy, human diseases, xenobiotics

## Abstract

Altered immune responses associated with human disease conditions, such as inflammatory and infectious diseases, cancers, and autoimmune diseases, are among the primary causes of morbidity across the world. A wealth of studies has demonstrated the efficiency of nanoparticles (NPs)-based immunotherapy strategies in different laboratory model systems. Nanoscale dimensions (<100 nm) enable NPs to have increased surface area to volume ratio, surface charge, and reactivity. Physicochemical properties along with the shapes, sizes, and elasticity influence the immunomodulatory response induced by NPs. In recent years, NPs-based immunotherapy strategies have attained significant focus in the context of cancers and autoimmune diseases. This rapidly growing field of nanomedicine has already introduced ~50 nanotherapeutics in clinical practices. Parallel to wide industrial applications of NPs, studies have raised concerns about their potential threat to the environment and human health. In past decades, a wealth of in vivo and in vitro studies has demonstrated the immunotoxicity potential of various NPs. Given that the number of engineered/designed NPs in biomedical applications is continuing to increase, it is pertinent to establish the toxicity profile for their safe and intelligent use in biomedical applications. The review is intended to summarize the NPs-induced immunomodulation pertaining to toxicity and therapeutic development in human health.

## 1. Introduction

**Figure 1:**
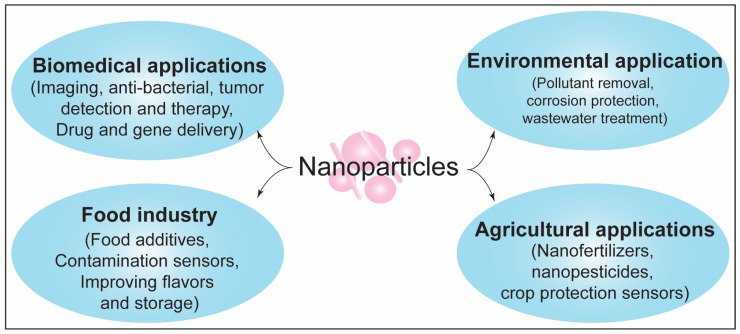
Wide application of nanoparticles in different sectors.

As the first responder of exogenous and endogenous stimulants, homeostasis and maintenance of the immune system are critical for organismal growth and survival. The exogenous insults/stress to the organisms consistently challenge the immune system. Environmental chemicals fall under one of the major categories of exogenous stressors affecting immune functions. These exogenous substances modulate immune responses in the exposed organisms, acting as immunogenic or immunosuppressants. Both scenarios converge on immunotoxicity. Constant exposure to xenobiotics also makes humans prone to other immune-related adverse conditions such as infectious diseases. One of the emerging categories of environmental chemicals is nanoparticles (NPs). NPs have at least one of the dimensions in nanoscale, i.e., <100 nm [1]. The high surface area to volume ratio in the nanoscale makes NPs highly reactive and catalytic. Industrial applications of NPs include food additives, cosmetics, medicines, agriculture, textiles, and electronics (Figure 1). Such a wide range of application has led to the release of NPs in the environment. Exposure to various form of NPs poses a risk to the environment and human health [2,3]. NPs can enter the cellular system through membrane invasion and make intracellular aggregates leading to physical damage to the lipids bilayer [4,5]. NPs can impair the redox signaling and result in cytotoxicity in the exposed systems cells [5,6,7]. Studies on different in vitro and in vivo systems have shown the differential toxicity profile of various NPs in immune cells [8,9]. Physiochemical properties associated with the nanoscale size of NPs and their potential to alter the various cellular and organismal responses, including immune response, have been harnessed for biomedical purposes, including immunotherapy, drug delivery, and vaccine development [10]. In the past two decades, toxicity assessment of NPs and immunotherapeutic purposing studies have run parallelly. Understanding the toxicity factor and modification of NPs is a promising approach to utilizing NPs in biomedical applications. Considering the advancement in nanotechnology and the growing application of NPs in the biomedical industry, the review aimed to stress the immunomodulatory potential of NPs in the context of toxicity assessment and therapeutic development. In this review, we begin by providing the simplified structure of the immune system and response machinery, followed by a discussion on xenobiotics-induced immunomodulation and associated human diseases. We further discuss the current state of knowledge about immunomodulation by nanoparticles in the context of their toxic potential and therapeutic implications.

## 2. General Structure of Immune System and Response Machinery: A Brief Overview and Methods

**Figure 2:**
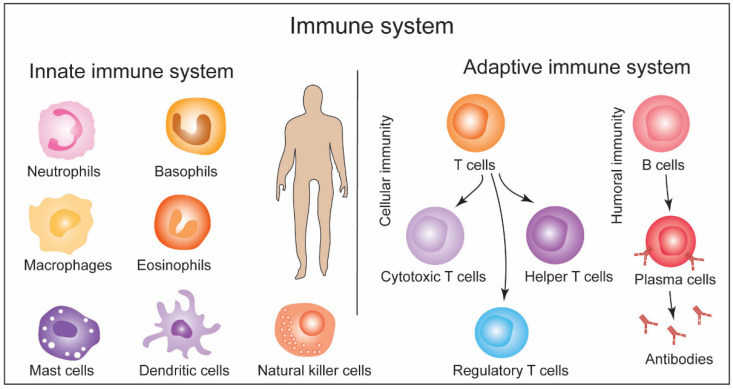
A simplified overview of immune system showing the components of innate and adaptive immune systems.

The immune system comprises cells, proteins, and chemicals and is categorized into the innate immune and adaptive immune systems. The innate immune system is an evolutionarily conserved first line of defense. It is composed of physical barriers (e.g., skin and mucus membrane) and cells including neutrophils, monocytes, mast cells, dendritic cells, basophils, macrophages, eosinophils, and natural killer cells [11]. The innate immune system responds to infection/pathogen by clearing them independently or informing the second line of defense, i.e., the adaptive immune system. The adaptive immune system responds to specific ‘non-self’ stimulants and is composed of T lymphocytes (T cells) and B lymphocytes (B cells). T cells include cytotoxic T cells, helper T cells (TH), and suppressor T cells/regulatory T cells (Treg cells), which are employed in cellular immunity. B cells produce antibodies involved in humoral immunity [11]. Immune response by these T cells and B cells is the second line of defense after the innate immune responses become insufficient in clearing the infection/pathogen. Cytotoxic T cells neutralize the infected cells as a cellular immune response. TH cells can release cytokines to facilitate the cellular immune response and stimulate naïve B cells. Treg cells suppress the overactivation of immune response by deactivating T cells and B cells. Some immune cells play a role in detecting harmful antigens and then report to the adaptive immune system. These are antigen-presenting cells (APCs). Upon the detection of pathogen/antigen, APCs phagocytose the pathogens to generate the fragments of the antigen, which are then loaded onto the major histocompatibility complex (MHC) and presented at the surface of APCs. Some innate immune cells, such as dendritic cells, and macrophages perform antigen-presenting functions [11]. Dendritic cells are present in almost all peripheral tissues, including skin, the lining of the nose, and the gastrointestinal tract. Upon activation, dendritic cells from their residing tissue niche to lymph nodes, and there these cells present antigens to the T cells and B cells. T and B cells are only activated upon recognizing epitopes presented by the APCs; otherwise they remain inactive or naïve. Naïve T cells expressing CD8+ recognize and bind APCs through antigen-associated MHC II molecules and become cytotoxic T cells. Naïve T cells expressing CD4+ bind APCs through antigen-associated MHC II molecular and become TH cells. The binding of T cells with APCs is facilitated by their T cell receptors (TCRs). TH cells that secrete cytokines to enhance the cytotoxic T cell functions are TH1 cells, while the ones that stimulate naïve B cells are TH2 cells [11]. Upon activation by TH2 cells, naïve B cells are differentiated into plasma cells that secrete antibodies. Naïve B cells harbor B cell receptors, i.e., immunoglobulins (Ig). Each Ig has two light and two heavy chains organized in a Y-shaped structure. Each of these chains possesses a variable region responsible for antigen binding and a membrane-bound constant region. TH2 cells release cytokines upon binding B cells to induce B cell proliferation. It leads to antibody production with identical antigen recognition patterns [11]. The schematic in Figure 2 represents a simplified structure and functions of the immune system. Immune response machinery briefly discussed above is modulated by xenobiotics, including NPs leading to immunotoxicity.

## 3. Immunotoxicity of Xenobiotics: Exposure Routes, Signaling Pathways, and Diseases Implications

The immune homeostasis against these xenobiotics is achieved by components of the host’s immune system. Failing to perform effective surveillance and elimination of these xenobiotics may lead to disease conditions such as immunodeficiencies, autoimmunity, and fibrosis, among others. In this section, we discuss the immunomodulation by xenobiotics, their routes of exposure, and implications for disease progression.

Exposure to immunotoxic xenobiotic substances, either occupationally or otherwise, can cause severe impairments to the immune system. This risk is further increased in infants, old individuals, or patients with primary immunodeficiencies. Environmental toxicants can also lead to chemical-induced immune dysfunction, often resulting in antibody formation and endotoxin sensitivity [12]. Evidences of neoplastic diseases, such as cancer, abnormal antibody production, prolongation of allograft rejection, reduced numbers of CD4+, CD+, and natural killer cells, have been reported in workers occupationally exposed to silica and asbestos [13,14]. The immunotoxic xenobiotics evoke inflammation by various mechanisms utilizing intracellular signaling pathways and intercellular mediators. The cells of the immune system, such as leukocytes and other plasma proteins from the blood, are then recruited to the site of inflammation to resolve it.

Xenobiotics may act directly on cells and tissues or via toxic intermediates and metabolites, such as small amines, peptides, enzymes, and lipids, causing oxidative damage and injury. They can also induce inflammation by interacting with cells of the innate and adaptive immune systems to facilitate the release of proinflammatory cytokines or increase hypersensitivity. The inflammatory response associated with xenobiotic substances can be local at the site of administration or a more systemic one depending on the extent of exposure. The intravenous administration may result in perivascular hemorrhage and release of proinflammatory mediators; oral administration can cause ulceration and inflammation, whereas inhaled xenobiotics cause necrosis and inflammation in the nasal cavity and airways. Inflammation caused by systemic toxicity can result in tissue and organ level damage, perivascular inflammation, and lesions. In addition, extracellular accumulation of uric acid crystals in response to xenobiotic administration can also cause NALP3 inflammasome activation and IL-1 secretion. Administration of monoclonal antibodies (such as anti-CD20 or anti-CD28) can stimulate T cell activation and cytokine release syndrome (CRS), leading to an increased secretion of proinflammatory cytokines TNFα, IL-1 β, IL-6, and IL-12. CRS is also one of the clinical features of COVID-19 infection [15,16]. In addition to the inflammation caused by the innate immune system, the adaptive immune response can also cause inflammation by facilitating the production of xenobiotic-specific antigens or general/tissue-specific hypersensitivity reactions.

Exposure to a specific class of xenobiotics can challenge the immune homeostasis and result in the development of autoreactivity. Several autoimmune diseases such as Rheumatoid Arthritis (RA), Systemic Sclerosis (SS), Systemic Lupus Erythematosus (SLE), Multiple Sclerosis (MS), and Anti-Neutrophil Cytoplasmic Antibody (ANCA) have been linked to occupational or lifestyle related exposure of xenobiotics. When an individual’s immune system starts to evoke a robust response against its own healthy cells and tissues, the condition is known as autoimmunity. The commonly targeted cells, tissues, and organs by xenobiotics to manifest autoimmunity include thyroid (thyroiditis), gastric parietal cells (gastritis), liver (autoimmune hepatitis and cholangitis), the β cells of the islets of Langerhans (diabetes), and steroid-producing cells in the adrenal and ovary (Addison’s disease), among others [17,18]. Many intrinsic and extrinsic factors contribute to the susceptibility to autoimmune diseases. The extrinsic factors include environmental, toxic chemicals, drugs, and microbes, whereas the intrinsic factors may include genetic makeup, age, and sex, among others. Although it is challenging to link exposure to xenobiotics to autoimmune diseases since the amplitude of autoreactivity differs, and the manifestation can take a very long time, attempts are being made by using various experimental models to decipher the mechanisms and contribution of immune components [17,18,19]. It can become further challenging as the same agent can induce a range of autoimmune disorders, whereas exposure to a variety of agents can produce a similar clinical feature [18]. *Drosophila* can be an excellent choice for studying xenobiotics-associated immune responses to expand the repertoire of animal models on disposal. Flies have most of the conserved signaling pathways involved in the immune response. They display context-specific cross-talks to execute a specific outcome [20,21]. In addition, they have been successfully implicated in studying immune responses against xenobiotics [22]. Xenobiotic exposure results in loss of immune tolerance, and autoantibodies development starts to occur. Depending on the xenobiotic, such as mercury or silica, it can develop preclinical or clinical manifestations [23,24].

Several studies have shown that the spontaneous or xenobiotic-associated autoimmune response shares a similar spectrum of immune components and mechanisms such as the involvement of cytokines IFN-γ and IL-1, IL-6, IL-17, complement regulatory factor CD55, and co-stimulatory molecules with a very little difference [25,26,27]. Once exposed to the xenobiotics, the tissue damage can cause the release of damage-associated molecular pattern molecules (DAMPs) or pathogen-associated molecular pattern molecules (PAMPs) if microbial contamination is involved. These are recognized by Toll-like receptors (TLRs), further amplifying the inflammatory response to the extent that it causes autoreactivity. Although the exact mechanisms of autoimmunity by xenobiotic exposure are still unknown, recent studies link a Pregnane X receptor (PXR) as a xenosensor that links toxic insults to PXR and nuclear factor-kappa B (NF-kB), Toll-like receptors (TLRs), and inflammasome components [28]. Further, NLRP3a-mediated inflammasomes are T regulatory cells also involved in heavy metals or drugs related to immunotoxicity and autoimmunity. When chronic inflammatory responses at the site of xenobiotic exposure lead to ectopic lymphoid structures (ELS) or tertiary lymphoid structures (TLS), it can allow maturation of B cells and the expression of disease-specific autoantibodies and hence adaptive immunity to develop into autoimmunity [29,30,31]. Another response to inflammation during xenobiotic exposure is fibrosis. Long-term exposure to fine particulate matter (PM2.5) can lead to chronic inflammation and pulmonary fibrosis [32]. It is characterized by the formation of excessive fibrous connective tissue owing to the depositions of extracellular matrix (ECM) components such as collagen. The ECM deposition is a common feature observed in many fibrotic diseases such as pulmonary and renal interstitial fibrosis, liver cirrhosis (LC), and myocardial infarction [33]. Both innate and adaptive components of immune responses are involved in the pathogenesis of fibrosis. Macrophages are reported to be involved in carbon tetrachloride (CCl4)-induced and NF-κB-induced liver fibrosis models [34]. The macrophage can be proinflammatory (M1) and anti-inflammatory (M2). Both of these cell types are involved in the development of fibrosis. Similarly, neutrophil-secreted elastase (NE) also has a pro-fibrotic role in asbestos-induced lung fibrosis; however, NK cells may have an anti-fibrotic role in liver fibrosis. The other cells of the innate immune system, such as γδT cells, can have a protective role in fibrosis models. Dendritic cells can also have a protective role in lung fibrosis, hence are an attractive candidate for immunotherapy against fibrosis [34]. In addition, the adaptive immune components can also have both pro- and anti-fibrotic roles. Many immune cell-mediated signaling pathways are also involved in the development of fibrosis. TGF-β, JAK-STAT, and mTOR signaling can promote disease progression in various fibrosis models [35,36,37,38]. A recent single-cell sequencing study on the PM-induced chronic lung injury mice model unveils the activation of the IL-17 signaling pathway in pulmonary fibrosis. The authors report that it was due to reduced recruitment of myeloid-derived suppressor cells (MDSCs) and downregulation of TGF-β signaling [32]. The xenobiotic stress, such as asbestos, is also reported to cause other non-pulmonary inflammation and diseases related to the peritoneum, gastrointestinal (GIT), and reproductive systems such as peritoneal and pleural mesothelioma, GI, colorectal, and stomach cancer, intratesticular mesothelioma, among others [13].

In the past decade, the effects of the structure–activity relationship and physicochemical properties of several of these emerging engineered nanoparticles on various immune system components have become an active area of research in nanobiotechnology. For example, some recent reports suggest that iron oxide nanoparticles can induce Th1-based immune response and monocyte-driven dysfunction of endothelial cells leading to atherosclerosis [39]. Another study reported that silver nanoparticles in zebrafish could lead to immunotoxicity, such as innate immune dysfunction, and affect the number and function of neutrophils and macrophages and the expression of immune-related cytokines and chemokines [40]. In the following sections, we focus on nanoparticle-induced immunotoxicity in various systems and immunotherapeutic uses of engineered nanoparticles.

## 4. Nanoparticles-Induced Immunotoxicity

**Figure 3:**
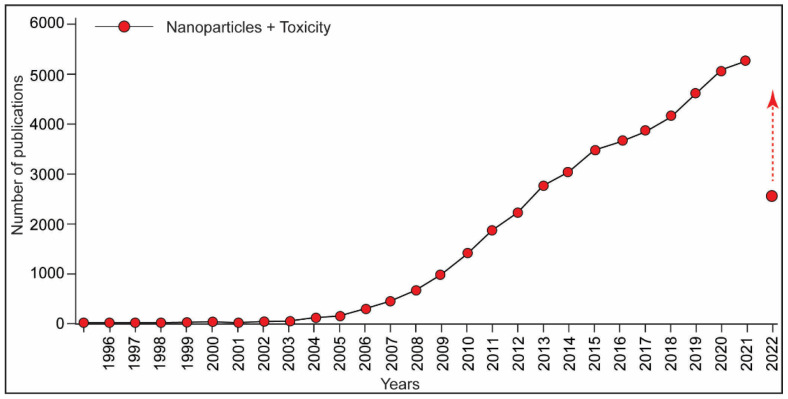
A graph representing the number of publications on assessment of nanoparticles toxicity. Data extracted using PubMed search engine (17 July 2022). Keywords were “nanoparticles” and “toxicity”. Note, this number includes the studies pertaining to the toxicity assessment of NPs. Not all the NPs examined in these studies were found to be toxic.

The broad scope of applications has placed engineered NPs in an emerging category of environmental chemicals/pollutants. The efficient use of NPs in the food and pharmaceutical industries has raised the concern for human health. This reflects with the increase in the number of publications about nanoparticle toxicity in the last decade (Figure 3). Studies about the toxicity assessment of NPs have demonstrated the adverse effects, including immunotoxicity in the exposed organisms, including humans [39,41,42].

**Figure 4:**
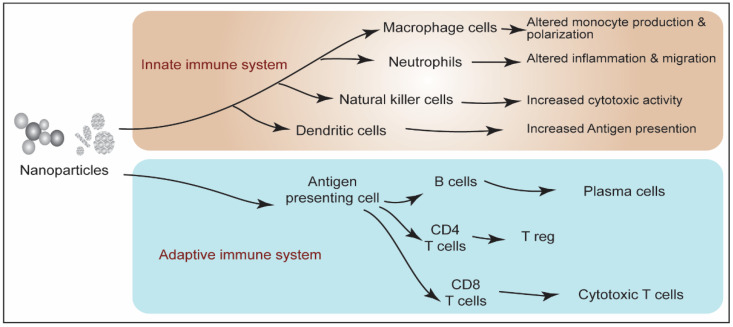
Schematic representation of innate and adaptive immune system response upon NPs interaction.

At the molecular level, NPs-induced immunotoxicity is mediated by ROS generation. Physicochemical properties of NPs facilitate the overproduction of ROS in the cellular system via different means, including the reaction of NPs with other biomolecules and impairment of the mitochondrial respiratory chain [43,44]. NPs alter immune system functions in the exposed organism either via hyperactivation of immunogenic response or by suppressing immune response induction. Both scenarios lead to immune function associated maladies/adversities. NPs can directly invade the immune cells and evoke cytotoxicity, which can compromise the immune response efficiency of the exposed organism. Both innate and adaptive immune components are equally affected by NPs exposure. Figure 4 represents NPs-induced responses in innate and adaptive immune systems.

The use of NPs in the food and pharmaceutical industries has raised the possibility of the unavoidable interaction of these NPs with the components of innate and adaptive systems. When NPs enter the body, they first interact with the innate immune system. The physicochemical properties of NPs such as shape, size, and surface modifications dictate how they interact with, evoke, or suppress the innate and adaptive response. In addition, these properties also govern the interaction of NPs with cells, receptors, and other components of the immune system and downstream signaling events. The NPs are internalized by cells of the innate immune system, such as macrophages and dendritic cells, by pinocytosis, clathrin/caveolar-mediated endocytosis, and phagocytosis. Usually, the smaller NPs are internalized by pinocytosis or endocytosis whereas the bigger NPs are engulfed via phagocytosis [45]. Further, the NPs are not inherently toxic; however, when bound to any carrier protein, they may be internalized by B cells. Following antigen presentations by B cells to T helper cells, this immunogenicity may lead to cytokine production by T lymphocytes and antibody production by plasma B cells [46].

### 4.1. Inorganic-Based NPs and Immunomodulation

Immunotoxicity has been assessed for all the four categories of NPs including inorganic NPs, carbon-based NPs, organic NPs, and composite-based NPs. Inorganic nanoparticles include metal and metal oxide NPs such as silver, zinc oxide (ZnO), and titanium oxide (TiO_2_) NPs. One of the widely studied engineered, inorganic NPs; silver NPs has been demonstrated to exert cytotoxicity in immune cells [47]. Silver NPs can penetrate the neutrophils and induce ROS overproduction leading to atypical neutrophil cell death [47]. Innate immune toxicity of silver NPs was observed in the zebrafish model. Silver NPs decreased the number of neutrophils and macrophages in the exposed zebrafish [40]. Mice-derived macrophages (RAW264.7 cells) and neutrophils (MPRO 2.1 cells) exhibited increased phagocytic activity and inflammatory response upon silver NPs exposure suggesting the immunomodulatory effect of these NPs [48]. A study on the rat model showed a dose-dependent reduction in the level of immunoglobulin G and M upon exposure to chitosan-coated silver NPs [49].

In addition to the use in medicine, titanium dioxide (TiO_2_) NPs are used in food additives and cosmetics and have shown a significant immunotoxic potential [50,51,52]. A recent report on primary peripheral human blood mononuclear cells (PBMC) showed an altered redox state and cytokine release against TiO_2_ NP exposure [53]. In this study, the embedding of mesoporous silica nanoparticles with TiO_2_ NPs (TiO_2_@MSN) showed reduced induction of immune response, suggesting that this modification can adjust the NPs-induced immune response of TiO_2_ NPs [53]. BSA-functionalized TiO_2_ NPs exposure to murine macrophage cells induced ROS generation, nitric oxide production, and antioxidants (e.g., SOD and Nrf2) depletion [54]. TiO_2_ NPs induced activation of Toll-like receptors and phosphorylation of p38MAPK and SAPK/JNK in these cells [54]. In a study in a mice model, TiO_2_ NPs induced immunotoxicity in lymphoid tissues and altered T cell and innate immune response via NF-kB-mediated MAPK pathways [55]. In rats, TiO_2_ NPs exerted immunotoxicity through an imbalance of TH1/TH2 cytokines in the respiratory system [56]. TiO_2_-NPs-exposed mice showed increased macrophage inflammatory protein (MIP)-1α, MHC, interferon-γ-inducible protein-10, and interleukin-13 [57]. Another commonly used NP used in the food industry, cosmetics, and medicines is zinc oxide (ZnO) NP [58]. In exposed C. elegans, ZnO NPs suppress innate immune response via SKN-1/Nrf and p38 MAPK signaling [59]. Exposed BALB/c mice exerted age-dependent induction of immunotoxicity as aged mice showed altered CD4 and CD8 cells, IL-6, TNF-α, and IFN- γ upon ZnO NPs exposure [60]. ZnO NPs exposed to Wistar albino rats displayed a signature of immunomodulation as evidenced by increased CD3, CD11b, TLR4, and TLR6 genes [61]. Immunomodulatory effects of ZnO NPs were also shown to be protective against other xenobiotics-induced immunotoxicity. A study in a rat model showed that simultaneous administration of ZnO NPs suppressed macrophage activity, IL-2, and IL-6 levels, and oxidative stress exerted by chlorpyrifos (organophosphate pesticide) exposure [62].

### 4.2. Carbon-Based NPs and Immunomodulation

Carbon-based NPs including quantum dots and graphene have been assessed for immunotoxicity potential in different model systems. Parallel to the biomedical application of cadmium selenide (CdS) quantum dots, toxicity assessment of these NPs has been reported in several model systems. In the exposed mussel Mytilus galloprovincialis, CdS quantum dots induced ROS-mediated cytotoxicity in hemocytes and gill cells [63]. In the exposed mice, CdS quantum dots altered the phagocytic activity of macrophages [64]. Recently, CdS quantum dots exposure was shown to enhance neuroinflammatory response via activation of NLRP3 in the exposed C. elegans, mice, and microglia cells [65]. Interestingly, the immunogenic responses evoked by the CdS quantum dots were rescued upon their modification achieved through ZnS conjugation. It supports that the adverse effects of NPs can be modulated by their surface modifications, including coating and conjugation. Furthermore, such data not only stress the use of toxicological assessment to unveil the toxic potential of NPs but also enhance our understanding of the specific type of modifications.

### 4.3. Organic-Based and Composite NPs and Immunomodulation

Graphene oxide (GO) NPs are also being used in medicine, drug delivery, biotechnology, and imaging [66]. RNAseq analysis of GO-NPs-exposed zebrafish (Danio rerio) larvae and adults showed a significant increase in the expression of immune genes [67]. In a study on the human acute monocytic leukemia cell line, GO NPs and vanillin-functionalized GO NPs (V-rGO) exposure caused increased ROS generation, LDH level, and lipid peroxidation along with decreased MMP and ATP levels [68]. It further stresses the understanding of the parameter for the NPs functionalization. Safe modifications of NPs for different applications can help risk management against exposure hazards.

Most of the organic-based NPs, such as dendrimers, liposomes, cyclodextrin, and micelle, along with the composite NPs, are found to be biocompatible and are being used in biomedical applications. Liposomes and dendrimers are the majorly utilized NPs in drug delivery and imaging that can induce hypersensitive reactions including stimulation as well as immunosuppression [69]. DOXIL, a pegylated liposomal doxorubicin showed adverse effects on macrophage population by impairing the phagocytic activity [70]. Dendrimer conjugates suppressed the level of proinflammatory chemokines (IL-8, MIP-1α, and MIP-1β) and cytokines (IL-1, IL-6, and TNF-) in human dendritic cells and macrophages, and increased the level of CD80, CD83, and CD25 molecules [71]. Recent studies showing the newly developed/designed different biocompatible composite NPs and their immunotherapeutic potential are discussed in the later section. Around fifty composite-based nanodrugs had been approved for cancer, and more are under investigation [72]. Notably, due to the safety issues, some of the nanodrugs were withdrawn [73]. Both organic-based and composite NPs require careful assessment for their immunotoxic effects as this would reduce the clinical trial failures and side effects.

**Figure 5:**
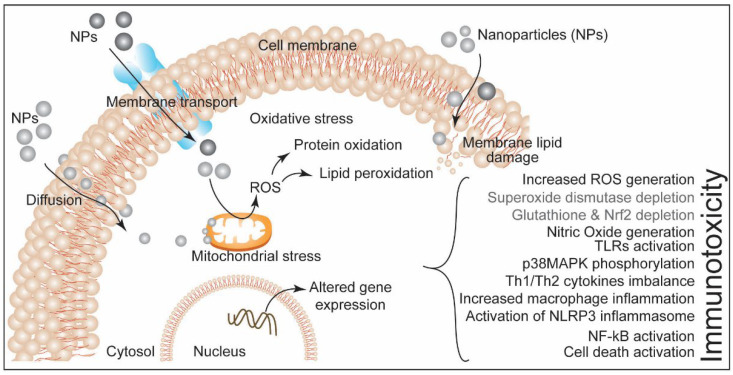
A schematic showing entry of nanoparticles in cellular system and the summary of cytotoxic effects leading to immunotoxicity.

Taken together, it suggests that engineered NPs induced immunotoxicity in the exposed systems. Altered ROS generation, cytokine release, and cell signaling pathways, including NF-kB and MAPK, are the major mediators of NPs-induced immunotoxicity. Modifications of the NPs, including conjugation, coating, or embedding, can potentially alter their adverse immune effects. In the biomedical field, such understandings, parameters, and properties of NPs can be utilized to modulate immune response as a part of therapeutic application. Schematic in Figure 5 represents the cytotoxic effects of NPs exposure leading to immunotoxicity.

## 5. Immunotherapeutic Aspects of Nanoparticles

**Figure 6:**
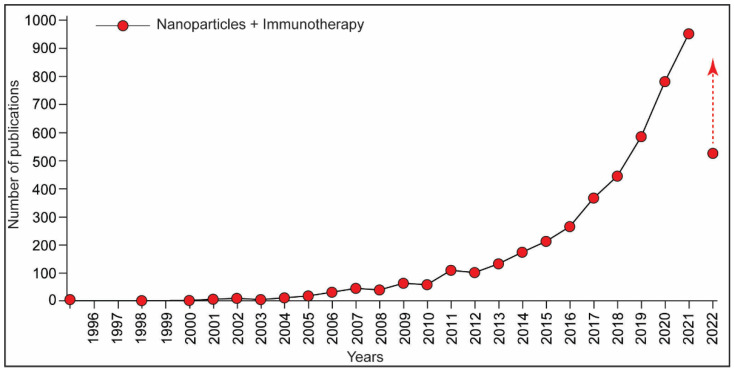
A graph representing the number of publications on nanoparticles-based immunotherapy. Data extracted using PubMed search engine (17 July 2022). Keywords were “nanoparticles” and “immunotherapy”.

Immunotherapy enables the treatment of diseases, such as cancers, using part of immune system machinery either through suppression or induction. Immunotherapy is categorized as active and passive. Active immunotherapy involves the adjustment of the intrinsic immune response, while passive immunotherapy deals with providing immune effector molecules/antigens to induce an immune response [74]. With the advanced technologies, immunotherapy is growing as a promising treatment approach for several human diseases, including cancers, AIDS, autoimmune, inflammatory, and infectious diseases. Various therapeutic modalities, including vaccines, cytokines, monoclonal antibodies, autologous T cells (CAR-T cells and TCR T cells), and designed small molecules (such as intracellular targets including COX2 inhibitors, TLR, and chemokine agonists) have been developed [75]. In the last five years, attention over the NPs-based immunotherapy has received significant interest as reflected with the relevant publications (Figure 6).

However, the implementation of immunotherapy approaches is insubstantial. The primary concern in immunotherapy implications is to provide an optimal immunotherapy benefit with proper kinetics to the specific site without inducing an adverse response to off-targets.

Engineered NPs can be adjusted for different parameters such as size, shape, and surface properties, enabling users to overcome the barriers in immunotherapy. Uptake, transport, and pharmacokinetics of NPs depend on their size [76]. The size of NPs is critical for their clearance. The migration of NPs also depends on the size. Smaller particles can migrate through diffusion, while the migration of larger particles needs a convective flow as well [77]. Multimodal in vivo imaging revealed that large NPs are extravasate with fast blood flow, suggesting that intratumoral distribution of NPs relies on local blood flow [78]. The size of NPs (0.1–10 nm) differentially influences their body biodistribution. The liver exhibits low accumulation of NPs with larger sizes, while the lungs exhibit increased accumulation with smaller sizes [79]. The shape of NPs affects the destination, biodistribution, and vascular transport [80]. The asymmetrical shape of NPs facilitates their internalization and distribution. The shape, size, and aspect ratio of NPs were shown to be important for their penetration in solid tumors [81]. Both the shape and size of NPs affect their immunogenicity. Size- and shape-dependent immunological responses were demonstrated upon exposing gold NPs of different shapes and sizes (spherical, rod, and cubic) to mice and RAW264.7 and bone-marrow-derived dendritic cells (BMDCs) [82]. Antigen-carrying spherical NPs induced the TH1 response pathway while rod-shaped NPs induced the TH2 response pathway [83].

**Figure 7:**
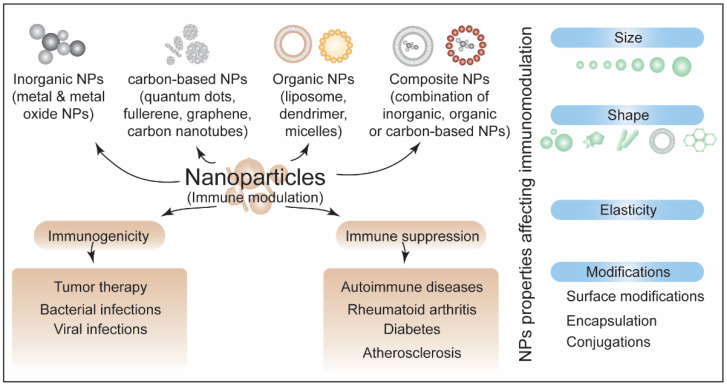
A schematic summarizing different forms of nanoparticles (NPs), aspects of immunomodulation, and the factors affecting these responses.

In addition to the size and shape, the surface charge of NPs has also been shown to influence immunotherapy application outcomes. Using pulmonary immunization with ovalbumin-conjugated cationic and anionic NPs, Fromen and colleagues reported that cationic NPs results in efficient humoral and mucosal immune response as compared to anionic NPs [84]. A similar study showed that positively charged gold NPs penetrate deeper into the skin than negatively charged NPs [85]. Ligand density of NPs is also reported to affect biodistribution and immune response. Liu and colleagues showed that differential distribution of lipid–calcium–phosphate NPs between hepatocytes and Kupffer cells was associated with the density of polyethylene glycol [86]. Polyethylene glycol-based hydrogel NPs were used with a range of elasticity moduli to examine the effect of NPs elasticity on their biological effects [87]. This study showed that softer NPs exhibited better circulation and efficient targeting as compared to harder NPs, suggesting that the elasticity of NPs can also influence the immunotherapy application. Taken together, the studies mentioned above suggest that engineering the properties of NPs can be utilized for immune response modulation. Figure 7 summarizes the immunomodulatory response of NPs and the factors affecting them.

**Figure 8:**
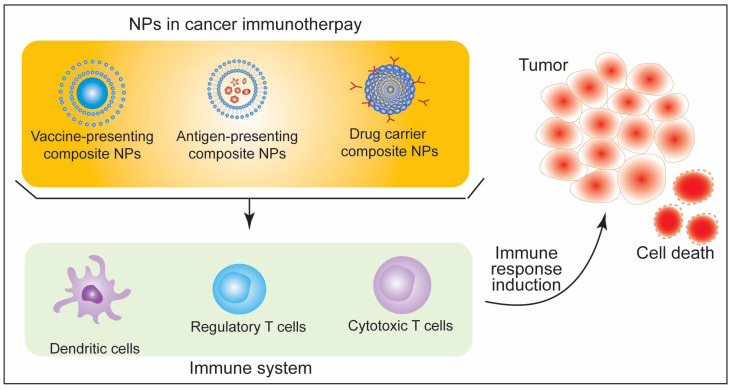
Schematic illustration of immune response induction against tumor.

Immunotherapy is widely implicated in the context of cancer treatment. In the last decade, there has been a rapid increase in the number of publications on nanoparticles and tumor immunotherapy. Cancer immunotherapy methods rely on blocking immune checkpoints, modifying T cell response, and characterizing the novel tumor antigens. An active cancer immunotherapeutic approach employs the self-immune response induction against the tumor cells through different means, including vaccination or targeting the antigen receptors. A passive immunotherapeutic approach employs cytokines, monoclonal antibodies, and lymphocytes that improve response to tumor cells [88]. Nanobiotechnology has advanced the scope of adjusting/engineering the unique properties of materials/particles obtained in their nano-dimensions. Various forms of NPs, such as gold NPs, liposomes, artificial exosomes, micelles, CNTs, and magnetic NPs, have been used as carriers in different strategies such as targeting dendritic cells, artificial APCs, targeting tumor-associated macrophages (TAM), myeloid-derived suppressor cells (MDSCs), and Treg cells [89,90,91,92,93,94]. Figure 8 illustrates the NPs-induced immune response induction against tumor.

**Figure 9:**
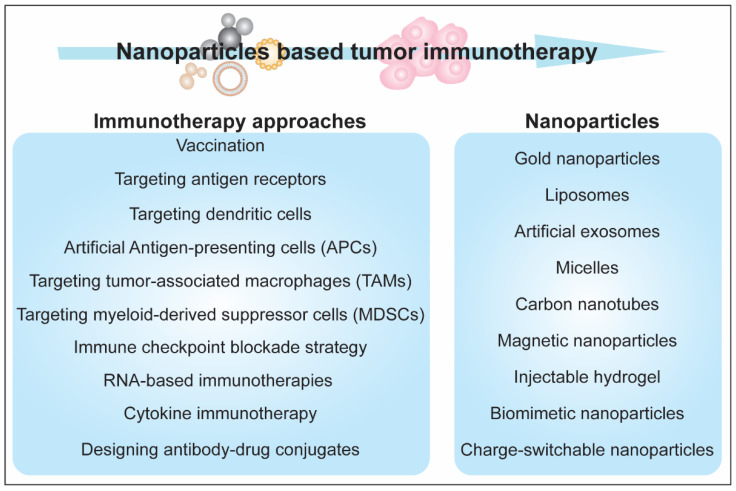
A summary of the strategies and nanoparticles being used/proposed for cancer immunotherapy.

Lately, studies on new engineering and designing strategies for NPs have evidenced the significant improvement in tumor immunotherapy. Zhang and colleagues designed an injectable hydrogel consisting of a lipid bilayer of a modified tumor cell membrane vesicle encapsulating axitinib and a cavity filled with 4-1BB antibodies and PF-06446846 NPs. This hydrogel was shown to reprogram T cell exhaustion and increase MHC I expression and T cell response via improved recognition of tumor cells [95]. The use of biomimetic NPs with the combination of chemotherapy and checkpoint blockade is another strategy for improved cancer therapy. Recently, an antitumor platform was designed using CT26 cancer cell-biomimetic NPs with a combined chemotherapeutic agent (RA-V) and BMS-202, a PD-1/PD-L1 blockade inhibitor and demonstrated that the combination of chemotherapy and checkpoint blockade enhanced the efficacy of immunotherapy against hypoxic tumor cells [96]. Another approach for cancer therapy is the use of charge switchable NPs. The size-reduced, change-switchable, and acidity-triggered NPs were designed to improve cancer therapy by blocking the indoleamine 2,3-dioxygenase 1 (IDO1) pathway [97]. Large-sized NPs with negative charge enhanced the tumor penetration and cellular uptake, while small-sized and positively changed NPs efficiently released mitoxantrone and IDO1 siRNA upon escaping lysosomes [97]. The charge-switchable NPs were designed to target tumor-associated macrophages [98]. This method induced an antitumor response and reversed the immunosuppressive tumor environment [98]. Modulation of natural killer cell responses has also been stressed for the potential NPs-based therapeutic implications in cancer therapy. Various functional aspects of natural killer cell response, such as activation and expansion of these cells and their migration to tumor sites, can be improved in NPs-based strategies [99]. Overall, the development of NPs-based redesigned therapeutic methods has dramatically upgraded the potential of immunotherapy against cancers. Figure 9 summarizes NPs-based strategies for caner immuno therapy.

In autoimmune disease conditions, the immune system responds against and attacks the host tissues [100]. The use of immunosuppressant drugs is a widely adopted treatment against autoimmune diseases. Suppression of immune response by NPs can be utilized as a therapeutic approach against autoimmune diseases. In line with this, a number of studies have evidenced the suppression of immune response by NPs in autoimmune conditions. Carbon nanotubes (CNTs) suppress T cell and natural killer cell activation [101]. Multiwalled CNTs internalized by APCs suppressed encephalitogenic Th17 cells [102]. Another class of carbon-based NPs, fullerenes suppress the allergic response in human mast cells and basophils [103]. Gold NPS was shown to affect the memory response of Bacille Calmette-Guérin (BCG)-primed human monocytes by suppressing the secondary inflammation [104]. Iron oxide NPs inhibited the IL-6 and IL-17 production in ovalbumin-sensitized splenocytes of BALB/c mice [105]. Treg is involved in the suppression of other immune cells and helps in relieving autoimmune disease conditions. NPs have been previously reviewed for harnessing Treg cells to suppress the immune response against autoimmune diseases [106,107,108]. It suggests the NPs-based immunosuppression as a promising approach for autoimmune disease.

The utilization of NPs has the potential to upgrade the efficiency of vaccines. One of the NPs-based treatment strategies for cancers and autoimmune diseases also includes the development of NPs-based vaccines [109,110]. NPs-based vaccines induce more robust T cell responses as compared to soluble vaccines. A study on pulmonary vaccination strategy demonstrated that using TLR agonist with antigen-loaded lipid capsules exerts high-frequency, long-lasting antigen-specific memory T cell responses at mucosal sites [111]. Another report showed that the antigen conjugated with polypropylene sulfide solid-core NPs induced a CD8 response while a polymersome-based watery core particle induced a CD4 response [112]. This suggests that the selection of vaccine core can be used to tune the T cell immune response. The porosity of the nanocarrier influences the immune response elicited by vaccines. Wang and colleagues made a sustained vaccine release system by entrapping bovine serum albumin (BSA) into the porous silica NPs and found increased titer of IgG and IgA upon oral immunization using silica NPs with large pores [113]. Using a chitosan nanogel-carrying ovalbumin antigen for their interaction and processing by dendritic cells, Thomann-Harwood et al. showed that surface presentation and vaccine cargo affect immune response [114]. It is evidenced that the understanding of the possible interactions and immune responses evoked through immunization of NPs-based model antigens can provide clinical benefits against different human diseases through the development of efficient vaccines.

Taken together, the studies summarized above provide mechanistic insights into NPs-induced immunotoxicity, and how they interact with cells and other components of the immune system and affect innate and adaptive immunity. This is achieved via either hyperactivation of immunogenic response or suppression of immune response induction. The examples discussed above indicate that the immune responses include increased ROS generation, superoxide dismutase depletion, NO generation, TLR, NF- κB, and NLRP3 activation. Since the physicochemical properties of NPs affect innate and adaptive responses, these properties can also be harnessed and tweaked to design effective immunotherapy.

## 6. Conclusions

Highly promising results in biomedical applications, including immunotherapy, are increasing the utilization of NPs-based therapeutic methods. Different means of administration of modified NPs-based medicines, including oral, dermal, and subcutaneous, have led to the reach and distribution of the NPs to almost every tissue. Although NPs have been demonstrated to solve the challenges/barriers in immunotherapy to an extent, it is important to explore all the possible factors affecting the efficacy and adverse response of the modified/newly designed NPs. A critical toxicity assessment of different forms of NPs using in vitro as well as in vivo systems is necessary. The lymph node proliferation assay (LPNA) and the plaque-forming cell (PFC) assays are the commonly recommended in vivo assays to predict the NPs toxicity. As per the International Organization for Standardization (ISO) standard 10993-4, regarding the assays for studying blood compatibility of medical devices, combined in vitro and in vivo safety assessment methods should be followed for the newly designed NPs. In addition, an assessment of NPs-induced immunotoxicity is recommended to be used on highly sensitive models such as pigs and dogs [115]. It also becomes pertinent to understand/explore the effect of NPs-based immunotherapy on immune system homeostasis. It is also important to study the potential effects of NPs-based immunotherapy on the signaling pathways associated with immune response. Successful and safe designing/engineering methods would allow the nanomedicine and NPs-based therapies to be the widely adopted treatment methods for various human diseases, including cancers, autoimmune diseases, and infectious diseases.

## Data Availability

Not applicable.
